# Supplementary material for: Examining the attitudes of sexually abused and non-abused individuals towards marriage in terms of ambivalent sexism
Source: PLoS One. 2023 Jul 7;18(7):e0288110. doi: 10.1371/journal.pone.0288110 (PMC10328297; doi:10.1371/journal.pone.0288110)
Supplement: Attachment — Submitted filename: Response to Reviewers.docx [file pone.0288110.s002.docx]

**RESPONSE TO REVİEWER(S)**

**Manuscript:** Examining the Attitudes of Sexually Abused and Non-Abused Individuals towards Marriage in Terms of Ambivalent Sexism, PONE-D-23-05383

Dear Editor/Reviewer(s),

Thank you for the valuable comments and suggestions. Your comment/suggestion is a valuable opportunity to further improve the quality of our work in terms of the technical content, novelty and quality of literary presentation.

| **Journal Requirements** | |
| --- | --- |
| **Comments** | **Responses** |
| 1. Please ensure that your manuscript meets PLOS ONE's style requirements, including those for file naming. The PLOS ONE style templates can be found at | Article revised according to PLOS ONE's style requirements |
| 2. Thank you for stating the following financial disclosure: | This statement was added in Cover Letter:  “The funders had no role in study design, data collection and analysis, decision to publish, or preparation of the manuscript. The authors received no specific funding for this work.” |
| 3. We note that you have indicated that data from this study are available upon request. PLOS only allows data to be available upon request if there are legal or ethical restrictions on sharing data publicly. | Data Availability statement was updated in cover letter: “The data that support the findings of this study are not publicly available due to their containing information that could compromise the privacy of research participants.” |
| 4. Please ensure that you include a title page within your main document. You should list all authors and all affiliations as per our author instructions and clearly indicate the corresponding author. | A title page including list all authors and all affiliations and other informations was added. |
| 5. Please review your reference list to ensure that it is complete and correct. If you have cited papers that have been retracted, please include the rationale for doing so in the manuscript text, or remove these references and replace them with relevant current references. Any changes to the reference list should be mentioned in the rebuttal letter that accompanies your revised manuscript. If you need to cite a retracted article, indicate the article’s retracted status in the References list and also include a citation and full reference for the retraction notice. | References in the text and in the reference list were reviewed and arranged according to the style of the journal. |

| **Review Comments to the Author** | |
| --- | --- |
| **Comments** | **Responses** |
| 1. The introduction section is written in great detail and it is suggested to write this section in a more concise manner and with more recent sources. | The introduction section is written in a more concise manner and with more recent sources. İn this context, non-essential sentences were deleted and updated references were added to the relevant sentences. |
| 2. In Table one and the first line, numbers 1, 2, and 3 represent which variables? | Variables represented by numbers (1,2,3) in Table 1 are written. |
| 3. In the conclusion section, are you sure about this statement? There are studies in this field that are below:  Ambivalent sexism in close relationships:(Hostile) power and (benevolent) romance shape relationship ideals  Ambivalent sexism and perceptions of men and women who violate gendered family roles | In the discussion section, the sentences stated by the referee were revised and current relevant research findings were added. |
